# Bi-directional hyperspectral reconstruction of cherry tomato: diagnosis of internal tissues maturation stage and composition

**DOI:** 10.3389/fpls.2024.1351958

**Published:** 2024-02-15

**Authors:** Renan Tosin, Mario Cunha, Filipe Monteiro-Silva, Filipe Santos, Teresa Barroso, Rui Martins

**Affiliations:** ^1^ Department of Geosciences, Environment and Spatial Planning, Faculty of Sciences of the University of Porto, Porto, Portugal; ^2^ INESC TEC - Institute for Systems and Computer Engineering, Technology and Science, Universidade do Porto, Porto, Portugal

**Keywords:** fruit maturation, latent structures, precision agriculture, spectral reconstruction, spectroscopy

## Abstract

**Introduction:**

Precision monitoring maturity in climacteric fruits like tomato is crucial for minimising losses within the food supply chain and enhancing pre- and post-harvest production and utilisation.

**Objectives:**

This paper introduces an approach to analyse the precision maturation of tomato using hyperspectral tomography-like.

**Methods:**

A novel bi-directional spectral reconstruction method is presented, leveraging visible to near-infrared (Vis-NIR) information gathered from tomato spectra and their internal tissues (skin, pulp, and seeds). The study, encompassing 118 tomatoes at various maturation stages, employs a multi-block hierarchical principal component analysis combined with partial least squares for bi-directional reconstruction. The approach involves predicting internal tissue spectra by decomposing the overall tomato spectral information, creating a superset with eight latent variables for each tissue. The reverse process also utilises eight latent variables for reconstructing skin, pulp, and seed spectral data.

**Results:**

The reconstruction of the tomato spectra presents a mean absolute percentage error of 30.44 % and 5.37 %, 5.25 % and 6.42 % and Pearson’s correlation coefficient of 0.85, 0.98, 0.99 and 0.99 for the skin, pulp and seed, respectively. Quality parameters, including soluble solid content (%), chlorophyll (a.u.), lycopene (a.u.), and puncture force (N), were assessed and modelled with PLS with the original and reconstructed datasets, presenting a range of R2 higher than 0.84 in the reconstructed dataset. An empirical demonstration of the tomato maturation in the internal tissues revealed the dynamic of the chlorophyll and lycopene in the different tissues during the maturation process.

**Conclusion:**

The proposed approach for inner tomato tissue spectral inference is highly reliable, provides early indications and is easy to operate. This study highlights the potential of Vis-NIR devices in precision fruit maturation assessment, surpassing conventional labour-intensive techniques in cost-effectiveness and efficiency. The implications of this advancement extend to various agronomic and food chain applications, promising substantial improvements in monitoring and enhancing fruit quality.

## Graphical abstract

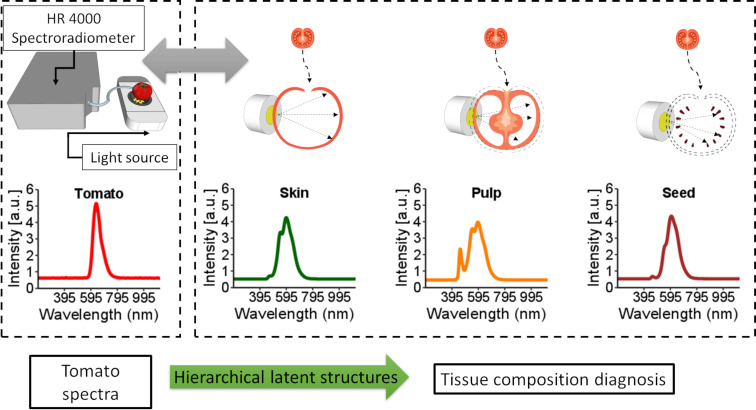

## 1 Introduction

Tomato is a climacteric fresh fruit composed of multiple tissues with diverse physical and biochemical compositions relevant to defining its quality through the food chain. The constitution of these tissues undergoes significant dynamic changes throughout the maturation process and in the post-harvest phase until the consumer such as the levels of antioxidants, lycopene, ascorbic acid, phenols and free radicals (Chandra and Ramalingam, 2011) and bioactive compound [e.g. flavonoids (Tamasi et al., 2019)].

The tomato fruit maturation process is marked by tissue specialisation, which promotes biochemical and physical changes in all tissues (Moco et al., 2007). During the ripening process, the tomato changes colour from green to red, resulting in morphological and biochemical modifications. The cultivar, environmental conditions (e.g., soil, light, temperature) and agronomic practices (e.g., irrigation, fertilisation) are essential factors that contribute to the tomato maturation process. For instance, in diverse regions, variations in antioxidants and phenols have been observed (Chandra et al., 2012). Under salinity conditions, morphological aspects such as size, water content, and colour undergo changes, impacting sugar content and acidity (Pascale et al., 2015). Additionally, genetic factors exhibit divergence even under identical conditions (Toor and Savage, 2005).

Several non-destructive techniques are available in the literature for characterising fruit maturation. However, none of these techniques can provide detailed information about the internal tissues of the fruit while also predicting its biophysical and biochemical characteristics efficiently. Although similar works approached the reconstruction of fruits using other techniques, such as electrical impedance to detect the tomato level of maturation (Verma et al., 2021), computed tomography in detect bitter pit in apples (Si and Sankaran, 2016) and X-ray in predicting the sugar content in kiwi fruit (Kanno and Kuroyama, 2020) and phenotyping charcteristics of seed in predicting the seed length, width, thickness, and radius of soybean and wheat with an accuracy ranging 80-96% (Liu et al., 2020), some of these techniques are expensive and not expedite for *in situ* measurements. Additionally, these methods are mainly used to identify physical damage, and none of them analysed each tissue individually or presented a qualitative approach (Donis-González et al., 2014). In biomedical science, non-destructive tissue characterisation (human and animal) has been developed through Vis-NIR spectroscopy, particularly for detecting tissue anomalies (Malone et al., 2014; Dahlstrand et al., 2019) and guiding the right incision during surgery (Vo-Dinh et al., 2010; Stelzle et al., 2012), which indicate that similar techniques can be applied to vegetation tissues.

Monitoring tomato precision maturation throughout the food chain is crucial for minimising losses within the food supply chain and improving pre- and post-harvest production and utilisation (Garcia and Barrett, 2006). From the high-tech horticulture point of view, monitoring maturation is essential to improve cultural practices, such as irrigation, fertilisation and canopy management, which are directly related to the pigments, organic acids and sugars in the tomato fruit (Bertin and Génard, 2018). In addition, to ensure that the seeds are fully developed for tomato seed production (Shrestha et al., 2016). They can guarantee that all tissues are well developed and that the gustative parameters favour the final consumer.

Traditional methods like chemical assays and chromatography used to characterise tomato biochemical parameters (e.g., lycopene) in different tissues are, in most cases, destructive, very expensive, non-adapted for small amount of tissues and time-consuming, which hinders the assessment of tomato quality parameters based on the composition of each tissue. Therefore, despite the high cost and time consumption of traditional analysis, alternative non-destructive techniques have been developed to assess tomato maturation and quality parameters, including colourimetric (Gómez et al., 2001), fluorescence (Wu and Wang, 2014; Konagaya et al., 2020), and Vis-NIR techniques (Torres et al., 2015; Zhu et al., 2015).

Vis-NIR devices have demonstrated outstanding potential for estimating fruit’s biochemical and biophysical parameters like lycopene and β-carotene (Tilahun et al., 2018), water potential (Tosin et al., 2021) and sugars and acids (Martins et al., 2022). Numerous studies on tomatoes have utilised Vis-NIR techniques to estimate various biochemical and biophysical parameters. These include soluble solid content (SSC) with reported R^2^ of 0.87 (Ecarnot et al., 2013), 0.60-0.77 (Torres et al., 2015), and 0.88-0.98 (Ding et al., 2016). The pH levels were determined with a R^2^ of 0.80 (Huang et al., 2018a), while colour attributes showed R^2^ values ranging from 0.91-0.99 (Ecarnot et al., 2013). Vitamin C content was estimated with a R^2^ of 0.67 (Azadshahraki et al., 2018), total acidity with R^2^ of 0.66-0.94 (Ding et al., 2016) and R^2^ of 0.91-0.98 (Najjar and Abu-Khalaf, 2021), and firmness exhibited R^2^ of 0.70-0.72 (Najjar and Abu-Khalaf, 2021). The analysis of β-carotene revealed R^2^ values of 0.77-0.88 (Tilahun et al., 2018), phenols showed R^2^ values of 0.76-0.98 (Ding et al., 2016), malic acid with R^2^ of 0.27-0.42 (Torres et al., 2015), citric acid with R^2^ of 0.66-0.94 (Ding et al., 2016) and lycopene with R^2^ values ranging from 0.45-0.75 (Clément et al., 2015), 0.73-0.83 (Ciaccheri et al., 2018) and 0.85-0.89 (Tilahun et al., 2018). However, tomato exhibits differences in the structural and biochemical characteristics of different tissues, which leads to significant ramifications in the absorption and scattering of light inside the tomato fruit (Skolik et al., 2019). These complex structures of tomato make it challenging to investigate the inner tissues through Vis-NIR spectroscopy and how they behave during maturation.

Spectral information about the inner tissues of fruits can be acquired using Vis-NIR data (Martins et al., 2023). Martins et al. (2022) demonstrated empirically that different grape tissues influence the whole fruit during the maturation process and that the concentration of pigments changes during maturation in various tissues. Obtaining spectral information of the internal tissues of fruits through Vis-NIR requires appropriate modelling techniques. Principal component analysis (PCA) is one such technique that uses latent variables (LV) to perform an orthogonal transformation of the original dataset onto a reduced subspace that is spanned by the principal components (Dahlstrand et al., 2019). Conversely, the combination of LV creates a superset, presenting a direct relationship with the original information. LV models can deal with significant and correlated variables (Trygg and Wold, 2003). Multi-block hierarchical PCA (HPCA) and hierarchical partial least squares (HPLS) are frequently used in chemometrics to deal with spectral information from different sensors and a batch of data (Mishra et al., 2021; Martins et al., 2023). Therefore, multi-block analysis can create a bi-directional reconstruction of whole tomato fruit from the skin, pulp, and seed spectral data.

Tissue reconstruction is based on hierarchical latent relationships between the spectral patterns of the observed tissues, providing details of the internal plant structures (Martins et al., 2023). This manuscript uses the term “tomography-like” to partly define the capacities of data-driven class reconstruction using hierarchical relationships. The practicality of bidirectionality involves connecting the principal latent space derived from tomato tissues to the entire tomato spectrum and executing the reverse process, which consists of breaking down the tomato fruit’s spectra into the spectra of its tissues, namely the skin, pulp, and seed. This methodology was recently applied to grapes, as demonstrated by Tosin et al. (2023), and it is expected to work in tomato, in which the tissue composition is different from grapes. The term tomography-like is debatable in that it only implies the resolution of the tissue image; here, it is aimed to provide a median spectrum of each tissue, given the fruit spectra in a non-destructive way. Once several spectra are taken from different positions on the fruit, a 3D resolved image can be reconstructed by relating the positions [x, y, z] and spectral gradients within internal tissues (Martins et al., 2022; Tosin et al., 2023). In this sense, this work can be considered ‘tomography-like’. Data-driven reconstruction does not use the same numerical approaches and solutions as the classical approaches but is entering several research areas due to their computational efficiency (Bar-Sinai et al., 2019; Martins et al., 2022).

Furthermore, spatially resolved tissue fruit composition is yet very complex to be obtained experimentally (Tosin et al., 2023). There are still very significant constraints at the level of analytical chemistry state-of-the-art on the quantity of sample that can be used to quantify parameters considered in today’s routine analysis, such as SSC and pigments. Therefore, metabolic or compositional imaging validation is still limited to the laboratory ground truth methods.

This research used a point-of-measurement (POM), where the light enters the fruit and has internal reflections, being only able to return to the spectrometer through a centre fibre optics pinhole, meaning that all light reaching the spectrometer interacts with the inner fruit’s tissues, maximising the spectral information on all internal tissues.

In multispectral or hyperspectral imaging, light is generally illuminated outside the fruit. This demonstrates that most light reaching the imaging sensor is reflected, non-absorbed, and carries little information about internal tissue composition.

Therefore, relationships with the recorded spectra using this method are generally limited to covariant information with pigmentation, which has the danger of quantifying through correlation and not due to the causal characteristic features present in the spectra. At present, it is believed that POM devices can be of more practical application to field studies than hyperspectral cameras.

Through multi-block analysis, this research facilitates the reconstruction of hyperspectral data by utilising information from individual spectra of tomato tissues, namely skin, pulp, and seed. This method enables the decomposition of the overall tomato spectrum into its constituent tissues, offering a bi-directional relationship. This study further provides a qualitative analysis of the tomato ripening process, elucidating the maturation levels in the skin, pulp, and seed at various developmental stages. By employing a POM sensing approach that maximises spectral information from internal tissues, the research addresses the limitations of traditional destructive methods, providing a non-destructive alternative for characterising tomato biochemical parameters. This contribution to the high-tech horticulture food supply chain can be used to ensure superior quality produce, reduce waste, enhance market value, and advance agricultural practices.

Therefore, three main objectives have been established in this work: i) to reconstruct the tomato hyperspectral data using information from the skin, pulp, and seed spectra through multi-block analysis; ii) to demonstrate that the spectral information of the entire tomato can be decomposed into the skin, pulp, and seed; and iii) to provide a qualitative analysis of the dynamics of the tomato ripening process, demonstrating the maturation levels in the skin, pulp, and seed, and how these tissues behave at different stages of the maturation process.

## 2 Materials and methods

### 2.1 Sampling and tomato properties

A total of 118 cherry tomatoes, freshly picked at several maturation stages, were promptly taken to the laboratory for analysis. Puncture force (N) and SSC (%) were performed after measuring the tomato spectra using a digital penetrometer (model PCE-PTR 200, PCE Group, D-59872 Meschede, Germany), registering the resistance force and maximal force until puncture and a hand refractometer Milwaukee model MR32ATC, with a scale range of SSC from 0 to 32.0%, respectively.

The tomato skin, pulp, and seeds were methodically extracted from each tomato (n = 118) and subjected to individual analysis to obtain their respective spectral records. Then, aliquots measuring approximately 0.5 cm² were taken using a lancet and deposited onto a glass microscope slide. The procedure involved peeling the tomato skin, slicing the pulp to a thickness of approximately 3 mm, and directly placing a single well-developed seed, selected from the various seeds present in the tomato, onto the microscope slide. This meticulous process ensured the separation of tomato tissues and allowed the acquisition of specific spectral data for the skin, pulp, and seeds.

The tomato process of maturation progresses from green to red and can be classified into six different colours: i) green, ii) breakers, iii) turning, iv) pink, v) light-red and vi) red (USDA, 1991).

**Figure 1:**
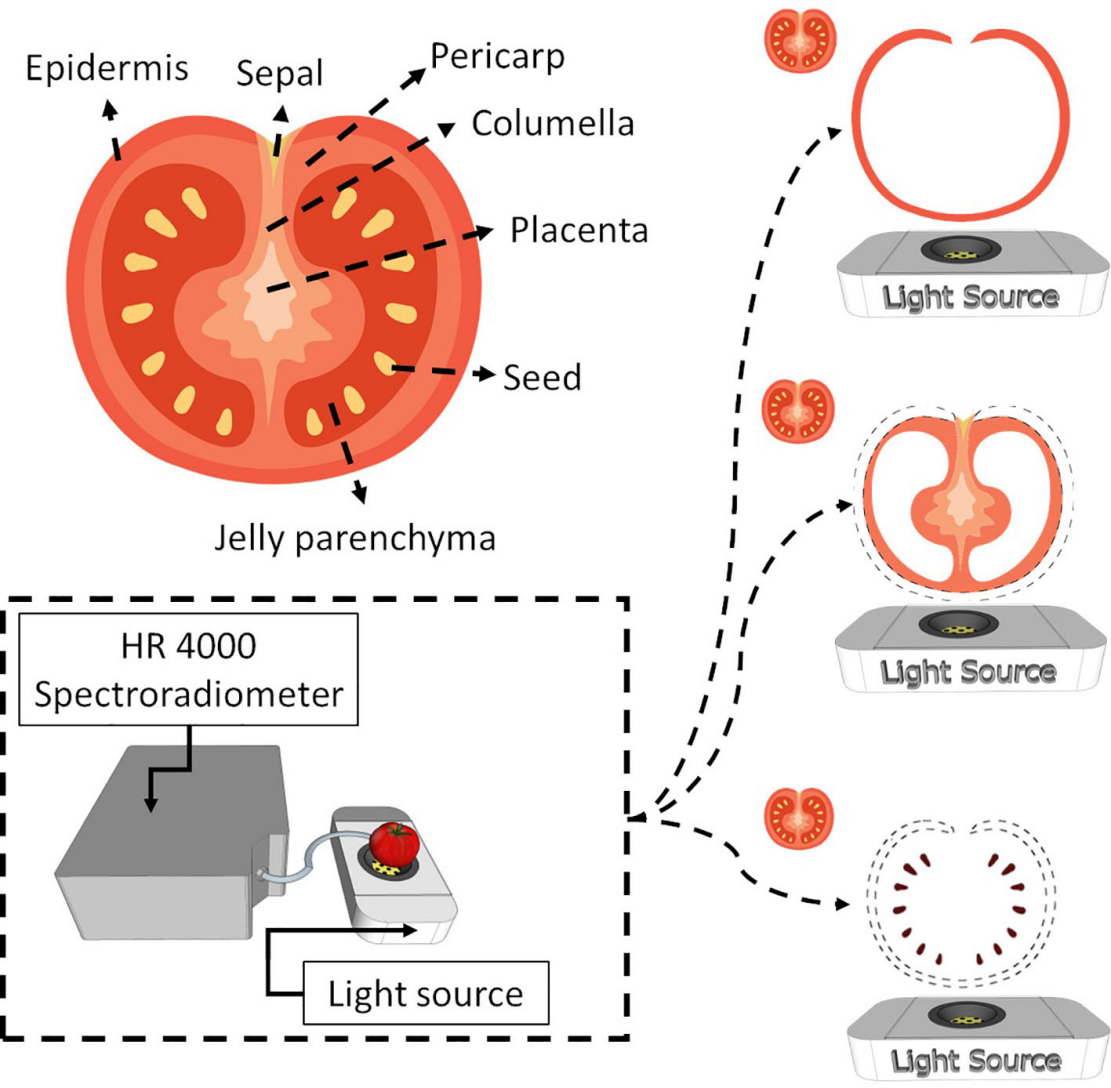
Internal tomato tissues and the spectroscopy system used to obtain spectral information from the entire tomato and the respective tissues (skin, pulp and seed).

Tomato is a complex fruit regarding internal tissues (**Figure 1**, **Supplementary Figure 1**). At the green stage of maturation, the tissues are not well developed, which makes tissue separation hard. Therefore, this work considered the epidermis as the skin, columella, placenta and pericarp as pulp and seeds as seeds (**Figure 1**). The jelly parenchyma and sepal were not considered in the analysis.

### 2.2 Spectroscopy

Tomato spectra were recorded with a white LED platform (**Supplementary Figure 1**). The platform comprises a reflection disk with a power LED (6500K, Philips SpotOn Ultra 69141/31/PH) at the bottom. The spectral range of the LED emits light from 380 nm to 780 nm. Therefore, LED spectra were used as a reference to check measurement and light emission stability. The tomato is placed above the LED, and the measurement is performed by collecting reflectance with a fibre optic probe (Ocean Insite). Skin, pulp, and seeds are placed on the microscope slide centred with the LED, and the reflectance probe also collects light. Spectra were recorded by a high-resolution spectroradiometer (Ocean Insite HR4000), which obtains information from 195.34 nm to 1118.33 nm; the integration time was optimised for each sample to maintain most of the spectra within the linear response.

After collecting all the spectral data, a logarithm multiplicative scattering correction (Martins et al., 2022) was applied to normalise and reduce the noise in the spectral information. The correction is a widely used method that addresses the issue of light scattering, which can distort the spectral signal and lead to inaccuracies in the measurements.

The logarithmic transformation helps to remove this scattering effect and improve the accuracy of the spectral data.

### 2.3 Hierarchical latent structures reconstruction

Latent structures are spaces obtained by matrix decomposition into their eigenvectors, a new basis where the contained information is projected. Latent structures provide a geometrical interpretation of the dataset and its samples by understanding their position on the eigenvector basis. Eigenvectors can be extracted with different properties, but one of the most common decompositions is PCA, where orthogonal eigenvectors are obtained by maximising the dataset variance, allowing to provide the interpretation of relevant variation. PCA can be obtained from the dataset X by singular value decomposition (SVD) after subtracting the mean of X : $X=USV^{t}$ ; where U is the left singular, S the singular values and $V^{t}$ the left singular. In PCA, the scores (aka latent structures) t are given by US , and the loadings (aka basis/eigenvectors) by $p^{t}$ = $V^{t}$ .

The geometry of information contained in X can be studied to determine what eigenvectors represent non-random information by performing randomisation tests (Martins et al., 2022), where the spectra dataset reconstruction can be decomposed into $X=tp^{t}+e$ , where e is random information, irrelevant for spectral reconstruction (Martins et al., 2023).

Let’s consider the corresponding database of tissue spectra: skin $X_{1}$ , pulp $X_{2}$ and seeds $X_{3}$ and their corresponding relevant PCA decomposition (**Algorithm 1**):


(1) $$X_{1}=t_{1}p_{1}^{t}+e_{1}$$

(2) $$X_{2}=t_{2}p_{2}^{t}+e_{2}$$

(3) $$X_{3}=t_{3}p_{3}^{t}+e_{3}$$

Where $t_{1}$ , $t_{2}$ , $t_{3}$ are the relevant latent features that reconstruct the original tissue spectra $X_{1}$ , $X_{2}$ , and $X_{3}$ , and $e_{1}$ , $e_{2}$ and $e_{3}$ discarded random spectral information. The latent information associated with each tissue can now be fused by determining the relevant common dimensions of their latent variance in geometry along each eigenvector.

Let’s take $t_{f}=\left[t_{1}^{i},t_{2}^{i},t_{3}^{i}\right]\;$ as the concatenation of the *i* dimension of $t_{1}$ , $t_{2}$ , $t_{3}$ , to be fused into a superset latent space T by finding the relevant information of each sub-level. The superset latent space can be determined by:


(4) $$t_{f}=T_{i}P_{i}^{t}$$

Being $T_{i}$ the superset latent information of the *i* dimension of the subsets (tissue spectra), and $P_{i}^{t}$ provide the contribution of each subset to the fused information $T_{i}$ .

If the information of $\left[t_{1}^{i},t_{2}^{i},t_{3}^{i}\right]$ has the same direction, $T_{i}$ will be described by a single eigenvector $P_{i}^{t}$ or a single dimension; otherwise, further relevant dimensions are added to $T_{i}$ .

The superset latent structure is constructed for each dimension of $X_{1}$ , $X_{2}$ , and $X_{3}$ , representing the relevant information of the tissue dataset, that is, the relevant characteristics from the skin, pulp, and seeds that relate to the observed tomato spectra.

### 2.4 Association and bi-directionality

The relevant features extracted from the sub-levels represented in the superset T have a similar latent structure to the direct PCA decomposition of the tomato spectra (Y) . By performing a PCA decomposition to Y , it is gotten $Y=UC^{t}$ ; where a direct association between the latent spaces T and U is expected ( T≃U ). One can expect that samples with similar composition and morphological characteristics will generate cluster aggregations in T and U , reflecting the different skin, pulp, and seed maturation state combinations (Martins et al., 2023).

**Figure 2:**
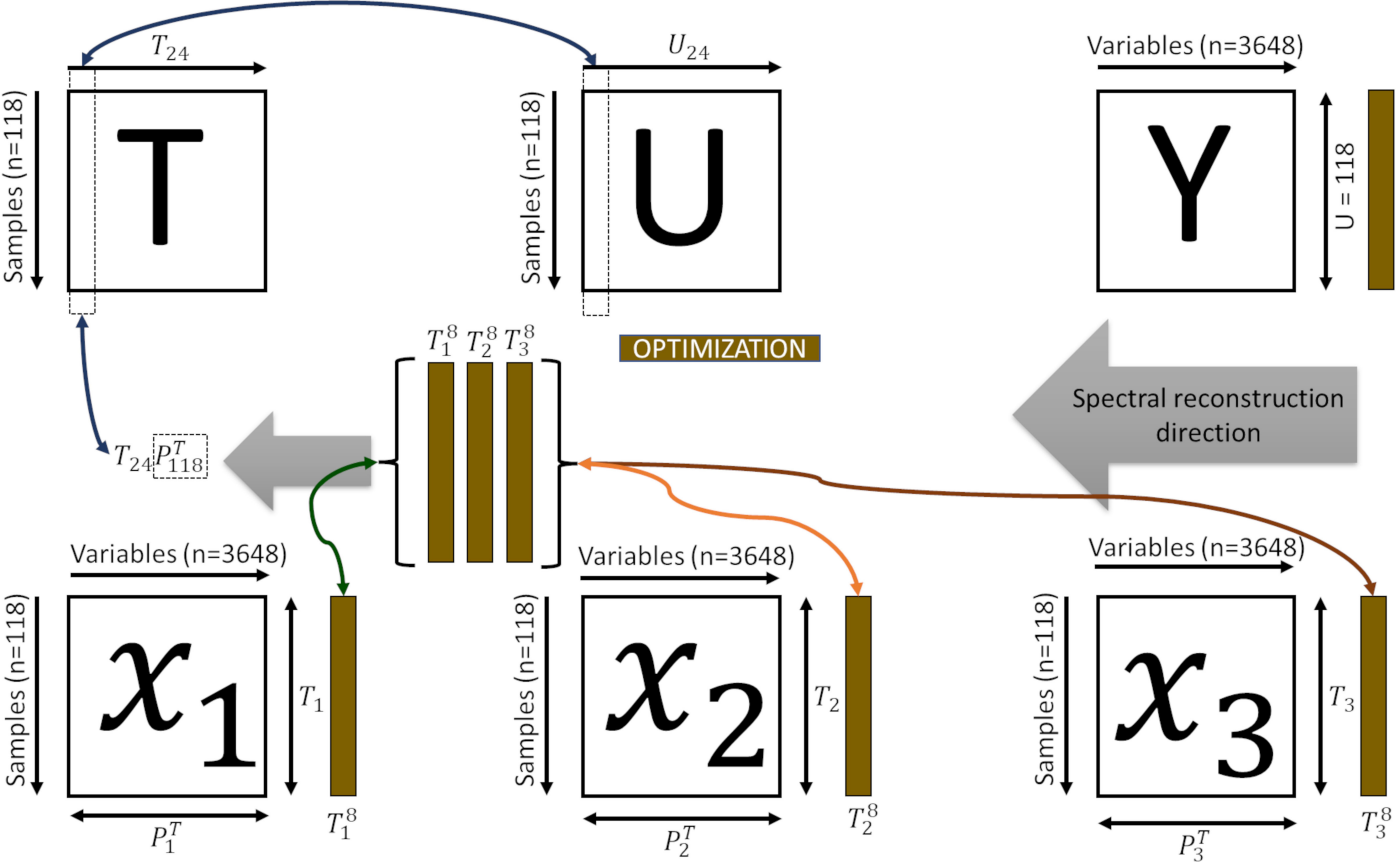
Representation of the bi-directional reconstruction process and decomposition of the spectral information. T is the superset latent space; ${\text{T}}_{24}$ the superset latent information of the dimension of the subsets ( ${\text{T}}_{1}$ , ${\text{T}}_{2}$ , ${\text{T}}_{3}$ ); U is the feature space of each subset; ${\text{U}}_{8}$ the superset latent information of the dimension of the subsets Y is the tomato spectra; ${\text{P}}_{118}^{t}$ is the contribution of each subset ( ${\text{P}}_{1}^{8}$ , ${\text{P}}_{2}^{8}$ , ${\text{P}}_{3}^{8}$ ); ${\text{X}}_{1}$ , ${\text{X}}_{2}$ and ${\text{X}}_{3}$ spectra of the skin, pulp and seed, respectively.

Therefore, one can establish a direct relationship between neighbouring samples in T or U , ensuring bi-directionality between the subsets $X_{1}$ , $X_{2}$ , $X_{3}$ and Y (**Figure 2**).

Inferring the internal tissues for a given unknown sample is performed by projecting the spectra Y into the feature space U , by U=YC , and finding the neighbouring samples ( k ) in this feature space. The k can be used to verify its propagation from T to the sub-level spaces $t_{1}$ , $t_{2}$ and $t_{3}$ , by reconstructing $t_{f}$ (**Algorithm 2**, Equation 4).

### 2.5 Validation

The hierarchical latent structure model was optimised and validated in a two-step approach: i. cross-validation (CV) to optimise the number of principal components (PC) of the sub-spaces $t_{1}$ , $t_{2}$ and $t_{3}$ and superspace T ; and ii. hold-out samples (HO) are used to test predictions and provide quantitative metrics.

CV is a test to the null hypothesis, and HO samples are double-check confirmations of the CV metrics. If the knowledge base is representative, any unknown HO or removed from the dataset, CV should provide statistically similar prediction metrics, proving the null hypothesis. By leaving samples out, CV provides the determination of the optimal error of each tissue reconstruction, $e=\left[e_{1}\;e_{2}\;e_{3}\right]$ (**Algorithm 3**, Equations 1–3). For each sample, the training set, the CV algorithm removes one sample (leave-one-out) for determining the error ( e ) for increasing the number of PCs of sub-space and superset. The optimal number of PCs is considered the one that provides minimal CV errors, preventing over-characterisation of random features at the sub-level passing into the superset. Suppose the training set is representative, CV and HO errors are expected to be similar. In that case, the model can efficiently reproduce the spectral information, and the null hypothesis is verified.

After reconstructing and decomposing the spectral data, a standardisation of the original, reconstructed, and decomposed spectral data was applied to mitigate the effects of signal intensity. Standardisation is a common practice used in data analysis to rescale variables with a mean of zero and a standard deviation of one. In this study, the formula ((x−mean)/sd) was used to standardise the spectral data. This method allows us to compare and analyse the spectral data more accurately, as it removes any differences in signal intensity that could impact the interpretation of the results.

The following metrics were used to benchmark spectral reconstruction: i. mean standard error (MSE) in counts/wavelength (nm), representing the average reconstruction error per wavelength (nm); ii. mean absolute percentage error (MAPE) in % wavelength (nm), representing the average bias per wavelength (nm); iii. Pearson’s correlation coefficient and the p-value were extracted to check for significant differences between the original and reconstructed datasets; and iv. Euclidean distances in T and U of CV and HO samples: This metric measures the knowledge-base representativeness and stability, which is important for evaluating the performance of the spectral reconstruction model.

Algorithm 1. Hierarchical latent structures algorithm
**Require**: 
$X_{1}$
, 
$X_{2}$
, 
$X_{3}$

**Ensure:** 
$j=argmin\left(X_{i}-t_{i}p_{i}^{t}\right)$



$\;X_{1}=t_{1}p_{1}^{t}$



$\;X_{2}=t_{2}p_{2}^{t}$



$\;X_{3}=t_{3}p_{3}^{t}$

**While** 
$j>j_{min}\mathrm{do}$
 

$\;\;\;\;\;\;\;\;\;\;\tau_{i}\leftarrow \left[t_{1}^{i}\left|t_{2}^{i}\right|t_{3}^{i}\right]$



$\;\;\;\;\;\;\;\;\;\;\tau_{i}=U_{i}S_{i}V_{i}^{t}$



$\;\;\;\;\;\;\;\;\;\;T_{i}=U_{i}S_{i}$



$\;\;\;\;\;\;\;\;\;\;P_{i}^{t}=V_{i}^{t}$

**end while**
**Output:** 
$T=\left[T_{1}\ldots T_{n}\right]$
; 
$P=\left[P_{1}\ldots P_{n}\right]$

Algorithm 2. Outer relationships for reconstruction 
Require: 
Y
, 
T

Ensure: 
$Y=UQ^{t}$



$U^{k}=Tb$




$T^{k}=Uc$



$\;\;\;\;\;Y^{k}=\left(Tb\right)Q^{t}$

**Output:** 
$Y^{k}$

Algorithm 3. Latent structures reconstruction algorithm
**Require:** 
Y
, 
T
, 
P
, 
$\left[t_{1},t_{2},t_{3}\right]$

**Ensure:** 
$j=argmin\left(X_{i}-t_{i}p_{i}^{t}\right)$



$\;\;\;\;\;Y=UQ^{t}$



     T=Ub



$\;\;\;\;\;[t_{1},t_{2},t_{3}]=T_{p}P^{t}$




$\;\;\;\;\;X_{1}=t_{1}p_{1}^{t}$




$\;X_{2}=t_{2}p_{2}^{t}$



$\;X_{3}=t_{3}p_{3}^{t}$

**Output:** 
$X_{1}$
, 
$X_{2}$
, 
$X_{3}$

### 2.6 Prediction of tomato quality

As a proof of concept, the organoleptic characteristics of tomatoes were predicted by comparing quantification results obtained from real spectral datasets with those obtained from reconstructed spectra using latent hierarchical structures. The visible range of the spectral data, which falls between 400-700 nm, contains valuable information related to pigments, specifically chlorophyll and lycopene content. Based on the findings of Ciaccheri et al. (2018) and Moco et al. (2007), the inferred chlorophyll content used the ratio of green (520-570 nm) to red (571-700 nm) spectral bands, and lycopene content used the ratio of red to green spectral bands. The results demonstrate that the reconstructed tissue spectra provide a good relationship to the estimates obtained with the fruit spectra, thus serving as proof of the principle of internal tissue quantification. Also, it presents other parameters obtained with the fruit, such as puncture force and SSC, which correlate with the reconstructed tissue spectra, further demonstrating the feasibility of the approach. Although there is a state-of-the-art, optimised ground truth method for measuring fruit composition at the fruit level for small fruits such as grapes (Martins et al., 2022; Tosin et al., 2023), recording that data would not provide significant advantages as it does not allow better tissue resolution quantification than the one presented in this work.

This investigation employed a computational approach to assess tomato pigment content in tissue reconstruction, driven by the need for a time-efficient evaluation of lycopene and chlorophyll. Wet lab analyses commonly used for larger tissue samples were unsuitable due to the small tissue quantities (around 0.5 cm²) involved in the spectral analysis (Tosin et al., 2023). Routine methods for these analytes require larger tissue quantities, hindering direct comparison with results obtained through computational methods (Clément et al., 2008; Tilahun et al., 2018). Sophisticated analytical methods for small tissue quantities are costly and impractical for evaluating a system at a low technology readiness level (TRL). Therefore, validating the results using expedited and cost-effective methods suitable for assessing the mentioned pigments and potentially other analytes is advisable. Adopting expedited approaches and avoiding expensive wet lab methods can validate the proof of concept without incurring high costs, facilitating a smoother transition for further system development and refinement.

The study employed a partial least squares (PLS) approach to predict the quality parameters of tomatoes. The dataset comprised 118 samples, divided into two sets: 70% (n=82) for training and 30% (n=36) for validation. This division of the dataset into training and validation sets ensures the utilisation of a significant portion of the data for model development while still allowing for robust evaluation and assessment of the model’s performance.

A robust validation technique, leave-one-out cross-validation (LOOCV), was employed to evaluate the model’s performance. This approach involved systematically excluding one sample at a time during the evaluation process, allowing for an accurate estimation of the model’s predictive ability and mitigating the risk of overfitting.

The determination of the optimal number of LV in PLS model was carried out through an assessment of root mean square error (RMSE) values. This integral step in PLS modelling aimed to minimise the RMSE, underlining its fundamental role in refining the model for superior precision and effectiveness in predicting outcomes. The selection of the ideal number of LV was strategically driven by the overarching goal of achieving the most accurate and reliable results, a chase evident in the search of minimised RMSE values.

Within the data-driven analysis, representativeness and hypothesis-testing principles serve as foundational pillars. Representativeness ensures that the dataset employed for training and validation accurately represents the entire population of interest. Hypothesis testing facilitates the formulation and evaluation of statistical hypotheses, guaranteeing the results’ reliability and significance.

By adhering to these principles, it is possible to construct robust models and generate reliable predictions in data-driven analysis.

Benchmarks were performed using the following modelling approaches: i. Similarity(Sim)-Euclidean distance as a metric of the spectral and compositional similarity between neighbouring samples in the feature space (e.g., FaChada et al., 2014); ii. Principal component regression (PCR) - where the latent structures of the sub-level spectra $\left[t_{1}\;t_{2}\;t_{3}\right]$ , superset T and tomato spectra U ; PLS maximises the covariance between the spectra X and tomato composition Y by determining the eigenvectors of $X^{t}Y$ (Martins et al., 2023). This method forces the latent structures of spectra and composition (PLS scores - U ) to be equal (NIPALS algorithm) (Ergon, 2009) for the determination of each correspondent basis $U^{t}$ and $Q^{t}$ (Geladi and Kowalski, 1986). It proceeds with deflation and sequential orthogonal eigenvectors of the remaining information in $X^{t}Y$ (Phatak and De Jong, 1997). The number of deflation or LV is optimised by cross-validation/hold-out samples with minimal predicted sum of squares (PRESS) (Krstajic et al., 2014). PLS uses an oblique projection to determine the $b_{pls}$ coefficients in $Y=Xb_{pls}$ (Phatak and De Jong, 1997; Ergon, 2009).

## 3 Results

### 3.1 Tomato tissue reconstruction

**Figure 3:**
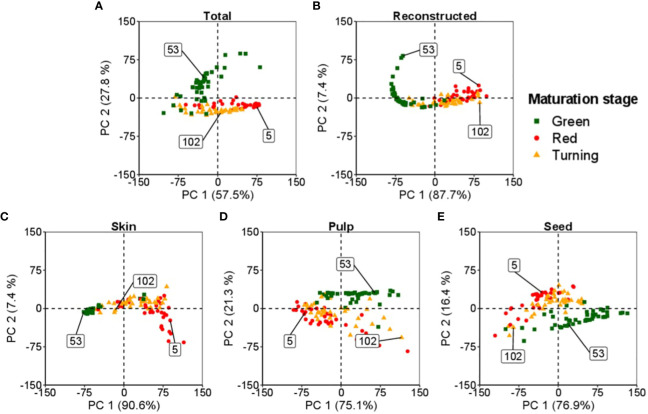
Spectral bi-directionality in reconstructing tomato components: skin, pulp, and seeds, at different maturation levels. The numbers in the box indicate the observations from the dataset for each maturation stage: ▪ (53) for green, ▴ (102) for turning, and • (5) for red. **(A, B)** represent the feature space for the total and total reconstructed spectra, respectively. **(C–E)** define the feature space of the skin, pulp and seed, respectively.

**Figure 3** presents an application of PCA to investigate the spectral data of tomatoes, including the entire tomato and its internal tissues (skin, pulp, and seeds). Each data point represents a unique spectral measurement obtained from an individual tomato. The primary objective of PCA is to identify a lower-dimensional representation of the data that captures the most important information.

In this study, the analysis classified the tomatoes into three maturation stages: i) green, ii) turning, and iii) red. A random sample from each maturation stage was selected to represent the feature space of these samples.

**Figure 3A** illustrates the comprehensive space occupied by the assessed tomatoes. Each tissue of the tomato, namely the skin, pulp, and seeds, possesses its distinct feature space, as illustrated in **Figures 3C–E**, respectively. PCA enables the decomposition of the space, resulting in individual spaces corresponding to each tomato fraction.

In **Figure 3B**, the plot demonstrates the reconstructed spectral data of the tomato obtained by combining the information from the skin, pulp, and seeds. The reconstructed data provides a condensed version of the original spectral data while preserving essential characteristics. It is also possible to reverse the process, enabling the data decomposition into the individual feature spaces of the tomato’s skin, pulp, and seeds.

The application of PCA in this study enhances the understanding of the tomato’s spectral data and internal structures. In addition, it provides insights into the relationships between different tomato fractions and their corresponding spectral properties, shedding light on the distinct characteristics of each tissue within the tomato.

**Figure 4:**
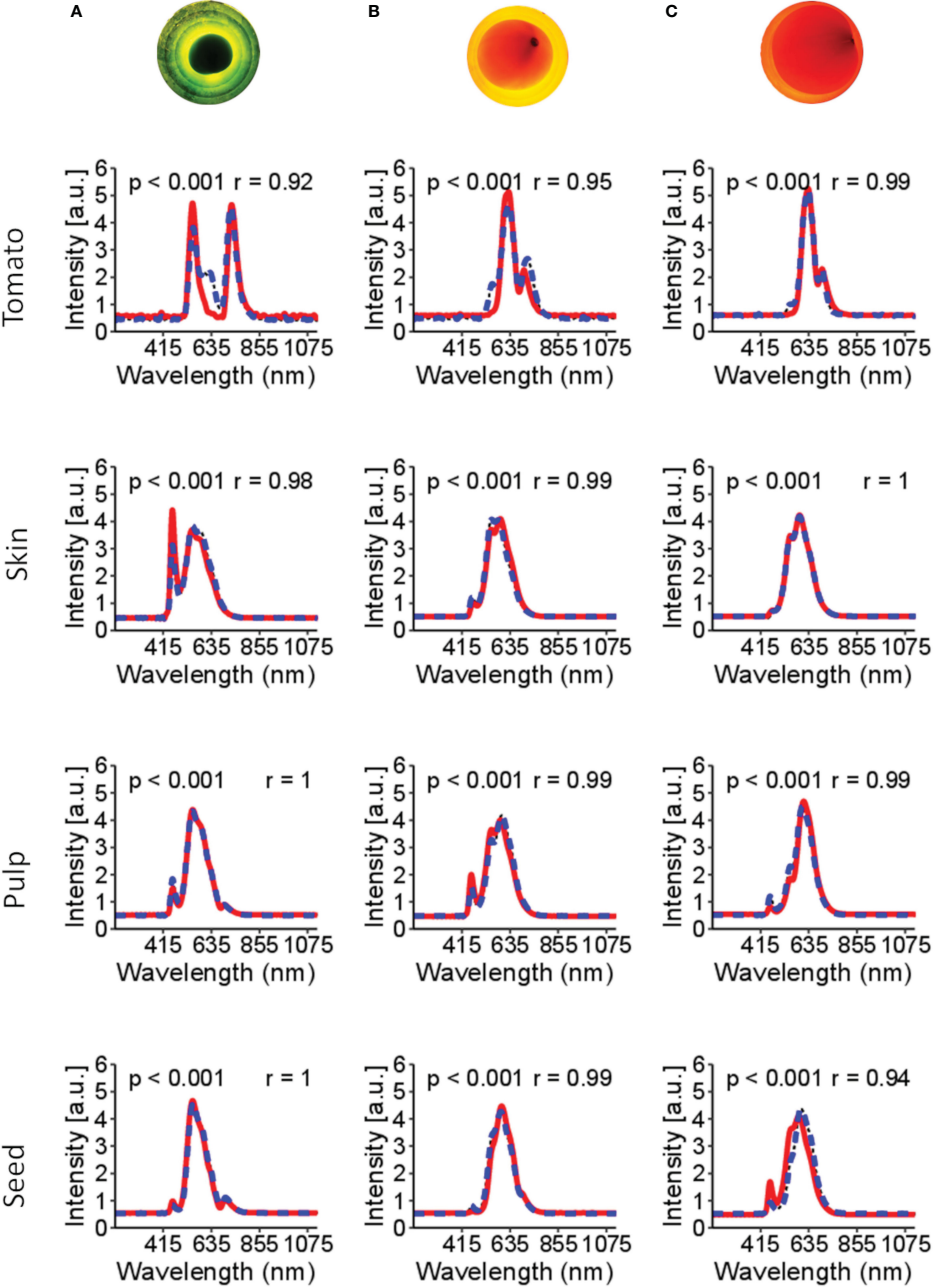
Spectral signatures of the tomato and the internal tissues. In the red line (—) is the original spectral signature, and in the blue dashed line (––) is the reconstructed spectral. Figures **(A–C)** are tomatoes subjected to LED light. Figure **(A)** is a tomato in the green stages of maturation; **(B)** tomato in the turning stages of maturation; **(C)** tomato in the red stage of maturation. The p-value< 0.001 indicates no significant difference between the original and reconstructed spectra. r is Pearson’s correlation.

**Figure 4** shows the spectral signature of tomato tissues at different stages of maturation: green, middle stage (turning), and ripened (red). The bi-directional spectral reconstruction (**Figure 2**) works better for internal tomato tissues than for the entire tomato (**Figure 4**, **Table 1**). In addition, during the experiments, it was observed that the light had more difficulty passing through the green tomatoes (**Figure 4**), which is a limitation in obtaining complete information about the internal tissues. It is suggested that at this stage of maturation, the tissues are not fully developed and contain a high concentration of pectin, which interferes with the optical properties of the tomato.

**Table 1:** Mean square error (MSE), mean absolute percentage error (MAPE), p-value and Pearson’s correlation coefficient (r) for the reconstruction of tomato spectra and decomposition of the entire tomato spectra into skin, pulp and seeds spectra.

Reconstruction	MSE(Count/wavelength (nm))	MAPE (%)	p-value	r
Tomato Spectra	0.30	30.44	< 0.001	0.85
Skin Spectra	0.04	5.37	< 0.001	0.98
Pulp Spectra	0.02	5.25	< 0.001	0.99
Seeds Spectra	0.03	6.42	< 0.001	0.99

Mean square error (MSE), mean absolute error (MAPE), and p-value< 0.001 indicate that there is no significant difference between the original and reconstructed spectral matrices.

The dynamics of maturation in tomato is shown in **Figure 4**. Based on these spectral signatures, different band peaks in the spectral signature are suggested to be related to tissue pigments (e.g., chlorophyll and lycopene). For example, green tomato presents higher signal intensity in the bands 460-500 nm and 660-700 nm, suggesting a correlation with chlorophyll content. Likewise, the 500-550 nm range of bands is more related to the carotene group, probably related to lycopene content in the more advanced stages of maturation.

**Table 1** presents a benchmark for spectral reconstruction and shows that the spectral reconstruction did not present significative differences (p-value< 0.001) over the original spectral data. However, compared with the respective tissues, the total spectral of tomato shows a higher MSE (0.30) and MAPE (30.4%) and a lower Pearson’s correlation coefficient (r=0.85; p-value< 0.001). Three leading causes can explain the low accuracy of the whole tomato: i) the green stage of maturation seems to be a hindrance for the light going through the internal tissues; ii) during the green stage of maturation, the tissues are not fully developed; and iii) the acquisition of the spectral information with an optical probe aimed at finding the best position to obtain the light signal through the internal tissues.

On the other hand, the decomposition of the whole tomato to predict the internal tissues worked better (**Figure 4**, **Table 1**). These results suggest that the spectral data of the tomato presented sufficient information on the internal tissues studied in this work.

The lower accuracy in the reconstruction of tomato is discussed in the next section.

### 3.2 Quality parameters evaluation

The original and reconstructed spectral information were used to predict the quality parameters of the tomato and their respective tissues. Overall analysis showed that the original and reconstructed spectral data were consistent and robust for predicting the quality parameters analysed (**Table 2**). Additionally, the different tissues of tomato could be used to predict the quality parameters assessed in this experiment. It is important to highlight that for SSC and puncture force, the entire tomato was considered for the measurement, and the different tissues of the tomato could predict these values for the whole tomato fruit. The results presented for chlorophyll and lycopene used an empirical dry lab method based on the spectral information of each tissue and the whole tomato, which helped to infer the pigment concentration in each tissue individually and in the entire tomato. Regression plots of the SSC (%), chlorophyll (a.u.), lycopene (a.u.) and puncture force for the skin, pulp and seed are presented in the **Supplementary Materials** (**Supplementary Figures 2–5**).

**Table 2:** Reconstruction benchmark of the tomato, skin, pulp and seed in predicting the soluble solid content (SSC), chlorophyll, lycopene and puncture force.

Property	Metric	Real dataset			
		Tomato	Skin	Pulp	Seeds
SSC (%)	r	0.99	–	–	–
	R^2^	0.98	–	–	–
	MSE (%)	0.85	–	–	–
	MAPE (%)	10.78	–	–	–
	LV	3	–	–	–
Puncture force (N)	r	0.98	–	–	–
	R^2^	0.97	–	–	–
	MSE (N)	2.94	–	–	–
	MAPE (%)	15.87	–	–	–
	LV	3	–	–	–
Reconstructed Dataset					
SSC (%)	r	0.99	–	–	–
	R^2^	0.98	–	–	–
	MSE (%)	0.86	–	–	–
	MAPE (%)	11.12	–	–	–
	LV	3	–	–	–
Chlorophylls (a.u.)*	r	0.95	0.99	0.99	0.99
	R^2^	0.90	0.99	0.99	0.99
	MSE (a.u.)	0.49	0.64	0.53	0.51
	MAPE (%)	51.32	6.01	10.06	13.51
	LV	2	2	3	2
Lycopene (a.u.)*	r	0.92	0.99	0.98	0.99
	R^2^	0.84	0.99	0.96	0.98
	MSE (a.u.)	0.62	0.64	0.73	0.57
	MAPE (%)	37.68	6.44	11.09	12.91
	LV	2	2	2	2
Puncture force (N)	r	0.90	–	–	–
	R^2^	0.95	–	–	–
	MSE (N)	3.84	–	–	–
	MAPE (%)	21.83	–	–	–
	LV	2	–	–	–

*Values computed with the spectral information (nm); the number of latent variables (LV) used in the partial least square (PLS). SSC and puncture force measurements were exclusively conducted for the entire tomato. Prediction based on individual tissues such as skin, pulp, and seeds is deemed impractical, given the integrated nature of these tissues. As a result, a dash (–) is denoted to signify the exclusion of these components in the predictive analysis.

The original dataset exclusively predicted SSC and puncture force for the entire tomato. Chlorophyll and lycopene content were predicted solely in the reconstructed dataset, as these pigments were estimated using real data, rendering their prediction in the original dataset nonsensical.

**Figure 5:**
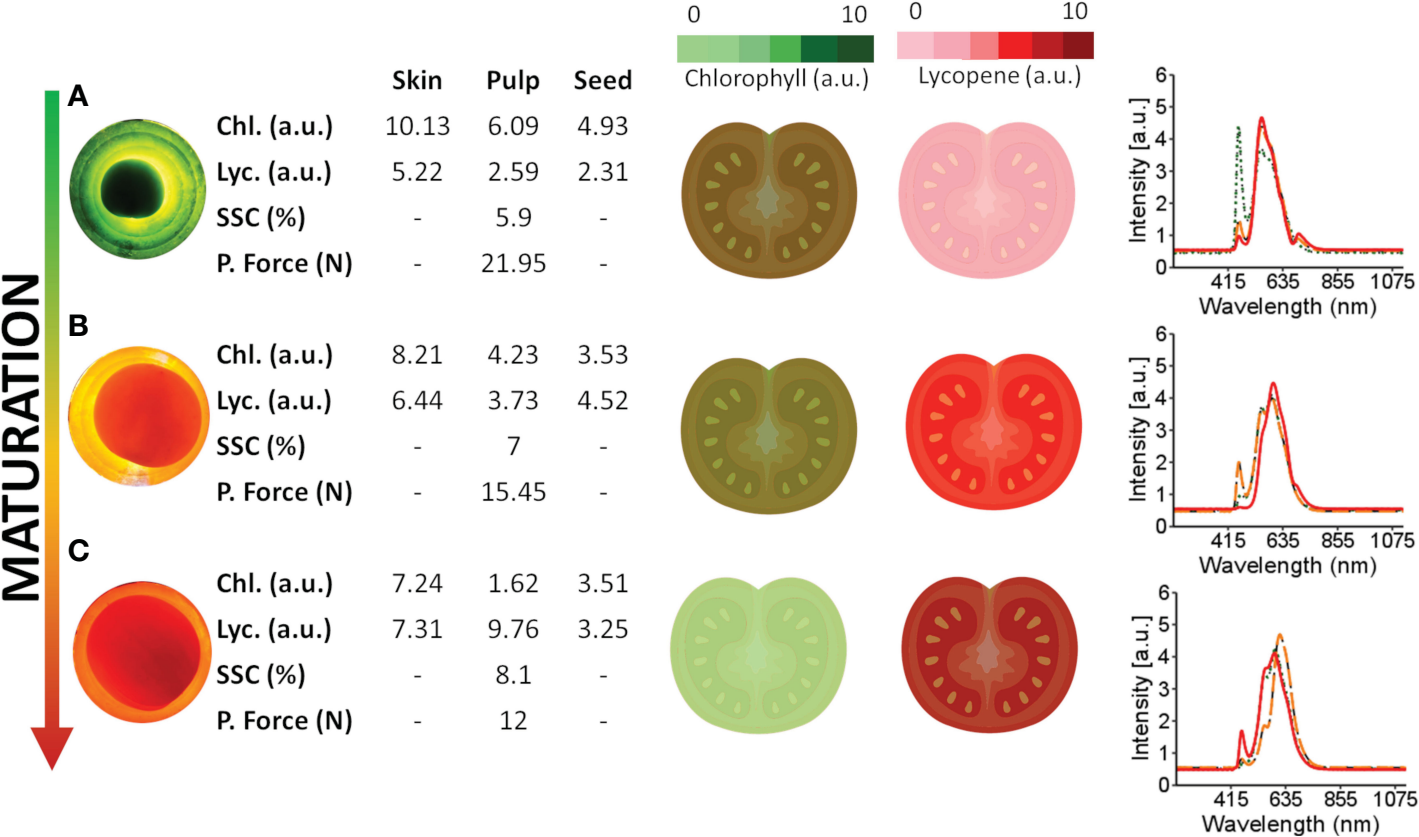
Spectral information obtained from tomatoes at different maturation stages and internal tissues and the dynamics of lycopene and chlorophyll during maturation. Chlorophyll content was empirically quantified, with light green representing lower levels and dark green representing higher levels. Lycopene levels were also quantified, with dark red representing lower levels and light red representing higher levels. **(A–C)** are red, turning and green tomatoes over the light source, respectively. The green spectra signature (·····) represents the spectral signature of the skin, the orange spectral signature (––) represents the spectral signature of the pulp, and the red spectra signature (—) represents the spectral signature of the seed.

**Figure 5** presents the changes in chlorophyll and lycopene concentrations in different tomato tissues during ripening. As the tomato ripens, chlorophyll concentration decreases while lycopene increases, as demonstrated by the spectral information and dynamic concentration data in **Figure 5**.

During the early maturation stage (**Figure 5A**), the tomato skin has a higher concentration of chlorophyll and a lower concentration of lycopene. Likewise, the pulp has a higher concentration of chlorophyll and a lower concentration of lycopene than other tissues of the tomato. This distribution of chlorophyll and lycopene is also reflected in the peaks observed in the tomato spectra. Specifically, peaks in the 460-500 nm and 670-700 nm range are associated with chlorophyll, while those in the 530-560 nm range are associated with carotenes, including lycopene. These wavelength assignments are drawn from the findings of Ciaccheri et al. (2018) and Moco et al. (2007).

During the ripening process, the peaks associated with chlorophyll decrease while those associated with lycopene increase, as shown in **Figure 5** for the tomato skin, pulp, and seed. These changes in pigment concentrations are responsible for the observed colour changes in the tomato from green to red as it ripens.

## 4 Discussion

This paper presents a disruptive methodology for the bi-directional reconstruction of whole tomato hyperspectral data and internal tissues (skin, pulp, and seeds). This non-destructive method can explain how different tomato tissues behave at various stages of the ripening process. As described in **Figures 1**–**3**, this work aims to bi-directionally reconstruct the spectral information of the tomato from the data of the skin, pulp, and seed and to decompose the information of the tomato spectra into the internal tissues. Although each tomato tissue presented a particular space (**Figure 3**), creating a superset with the scores of each tomato fraction made it possible to reproduce the entire spectral tomato (**Figure 4**). The most important LV of each fraction formed the superset used to reconstruct the tomato spectra. Through hierarchical PLS, the tissues could predict the entire tomato spectral data. The decomposition of the whole tomato data and the same number of LV combined in the hierarchical PLS can predict the tomato tissues. The literature reports that the tomography-like approach (Martins et al., 2023) successfully worked in grapes (Tosin et al., 2023), and the results of this paper support that it can be applied to aqueous fruits like tomato. Due to the complex nature of tomato maturation and the diverse biochemical compositions of its internal tissues (Skolik et al., 2019), encompassing skin, pulp, and seed, this work presents a technique for the bi-directional spectral reconstruction of tomatoes using Vis-NIR data.

The literature offers several methodologies (Mishra et al., 2021) that could be used for spectral reconstruction. For instance, O2-PLS (Trygg and Wold, 2003) and OnPLS (Lofstedt et al., 2013) utilise spectral data’s local and global joints, bioheat models (Alzahrani and Abbas, 2019; Marin et al., 2021) could be adapted to predict the internal tomato tissues and adaptive neuro-fuzzy inference system (Abdullahi et al., 2021; Abdullahi et al., 2022), a hybrid computational model that combines the adaptive capabilities of neural networks with the interpretability of a mathematical framework that deals with uncertainty and imprecision in decision-making. However, these methods are not hierarchical and do not allow for convolution and fusion of information in a superset or deconvolution in a reverse way. Similarly, advanced approaches such as deep learning (DL) can deal with complex data (Mishra et al., 2022) and reconstruct the whole tomato with spectral information from the different tissues. Nevertheless, the decomposition of the tomato spectra into the spectral data of its tissues becomes even more complex and challenging. Nonetheless, the results presented in **Table 1** show that hierarchical PCA combined with PLS can effectively perform bi-directional modelling with the directions X−Y and Y−X , and remove orthogonal data between Y and X , facilitating the reconstruction of the tomato and its internal tissues.

Owing to the multiple internal tissues that compose the tomato fruit, it faced a challenge in reconstructing the complete spectral information of the tomato (**Figure 4**). Furthermore, separating and identifying different tissues in green tomato are difficult because green tomato presents more fibre concentration (Chandra and Ramalingam, 2011) and pectin (Moco et al., 2007; Huang et al., 2018b). As a solution, all internal tissues (except the jelly parenchyma) were considered the pulp. Nevertheless, green tomatoes have less interest when compared with more advanced stages of maturation.

Spectral data acquisition encountered a few challenges related to the position of the tomato in the system used to obtain spectral data. First, green tomato is opaque, limiting light’s ability to pass through (**Figure 4**). This limitation requires the optical fibre probe to be well-positioned to obtain more light signals. However, searching for light using the probe may not obtain a signal from all internal structures, which limits tissue reconstruction. A similar effect was observed in matured tomato. Depending on the probe position, some internal tissues may not be assessed, or less information may be obtained. Second, the tissues considered as the pulp will affect the reconstruction of the entire tomato.

During the data acquisition of the entire tomato, almost all the internal tissues are expected to be evaluated using a fibre probe. However, to provide detailed information in the superset utilised to reconstruct the entire tomato, it may be necessary to individually assess each tissue considered as pulp and examine their specific details. Therefore, the errors observed in **Table 1** and **Figure 4** indicate less accuracy in the reconstruction of the entire green tomato and less in the other tomato fractions of the matured tomato. Nevertheless, this method can reconstruct the Vis-NIR spectra of tomato and internal tissues and be used for different purposes.

This work assessed the SSC, chlorophyll, lycopene, and puncture force of the tomato using the original and reconstructed spectra for each fraction of the tomato (**Table 2**). The SSC and puncture force were assessed in the entire tomato, and the spectral data of each fraction were considered in the modelling. Chlorophyll and lycopene provided spectral information for each tissue and empirically demonstrated the concentration of each pigment in the tissues. The original and reconstructed spectra results were very similar for reconstructing the entire tomato and the reconstructed spectra (**Table 2**).

Among the quality parameters assessed, the SSC results were the most stable and robust for the original and reconstructed spectra. Nevertheless, it is essential to highlight that the different tomato tissues present distinct SSC concentrations, and the individual tissues could have been assessed to predict the concentration of SSC in each fraction. For chlorophyll and lycopene, the ratio of the zones of the spectrum empirically demonstrated the different concentrations of pigments in the distinct tissues (**Supplementary Figures 2–5**). Considering all the tomato tissues, the skin is the part that is indicated to have more concentration of chlorophylls. This study reveals that the skin has the highest concentrations of chlorophyll and lycopene, except during the red maturation stage. These results align with existing literature, particularly Chandra et al. (2012), highlighting the skin’s elevated lycopene levels compared to pulp and seeds. However, in terms of chlorophyll content, the skin ranks as the second lowest tissue during maturation, as observed by Moco et al. (2007). The qualitative approach to classifying internal tomato tissues throughout maturation may contribute to these variations compared to the quantitative methods in the cited literature.

Consistent with prior research (Moco et al., 2007; Chandra et al., 2012), this paper reveals that the skin exhibits a higher concentration of lycopene, as shown in **Supplementary Figure 3**. Puncture force analysis indicates elevated values in green tomatoes, probably attributable to heightened fibre and pectin content. This coincides with lower levels of SSC and lycopene, alongside increased chlorophyll concentrations (Moco et al., 2007; Huang et al., 2018b).

The temporal dynamics of maturation across distinct tomato tissues are demonstrated in **Figure 5**, where chlorophyll concentrations, particularly in the skin, are higher in green tomatoes (**Figure 5A**). Conversely, matured tomatoes (**Figure 5C**) tend to exhibit increased lycopene concentrations, mainly in the skin. Considering the spectral signatures of the tomato fractions (**Figure 5**), further studies can be conducted to determine the type of information that can be extracted. Empirically assessing the full tomato spectrum (**Figure 5**), two bands peak near 500 nm and 690 nm in green tomato, probably related to chlorophyll (Ecarnot et al., 2013; Huang et al., 2018b). In the spectral skin signature, a similar peak (near 490 nm) in the green tomato may be related to chlorophyll a. When the pulp and seed were analysed, the same peak (near 490 nm) was present but with less intensity, suggesting a lower chlorophyll concentration in those tissues. The peaks near 550 nm are related to the carotene group (Ciaccheri et al., 2018), especially the lycopene concentration. For matured tomato, these picks present more intensity in the full tomato spectra, skin, and pulp, suggesting a higher concentration of lycopene when compared with less mature tomatoes.

Vis-NIR data can enhance the efficiency and quality of crop production by providing valuable information for optimising various agricultural practices, such as irrigation, fertilisation, and pruning (Xia et al., 2021). Furthermore, by leveraging this data, crop growers can gain precise insights into the maturation process of fruits, which is influenced by a range of biotic and abiotic factors, including diseases, water availability, temperature, and light intensity.

The methodology presented in this paper has the main advantage of providing more accurate and detailed information about the internal structure of the fruit as a tomography-like system when compared to the whole-fruit measurement by using hyperspectral or multispectral data (e.g., Mishra and Woltering, 2023; Mishra et al., 2023) that obtain majority external information. The tomography-like presented in this paper allows for the assessment of individual tissues, facilitating the acquisition of more accurate and detailed information about the internal structure of the fruit (Martins et al., 2023). **Figure 3** represents the feature space of the entire tomato and the skin, pulp and seed, where the different maturation stages occupy distinct feature spaces. In contrast, traditional whole-fruit measurements often lack precision in providing insights into the inner tissue of the fruit. Also, the methodology presented in this paper can lead to more accurate predictions of the internal tissue properties and better quality control. It can also enable a more specific and targeted analysis of internal tissue properties by offering comprehensive and high-dimensional data. These data provide more detailed information about the fruit’s internal structure and can also be utilised to determine additional components beyond those presented, particularly in supporting metabolomic studies. This knowledge can help to fine-tune agricultural practices and mitigate potential risks, ultimately leading to improved crop yields and higher-quality produce.

This paper presents a novel technique for determining the quality parameters SSC (%), chlorophyll (a.u.), lycopene (a.u.) and puncture force (N) of fruits using visible and near-infrared (Vis-NIR) spectroscopy of their skin, pulp, and seed. The approach builds upon a growing body of research demonstrating the ability of hyperspectral sensors to measure a wide range of quality parameters non-destructively and accurately in crops (Martins et al., 2022; Tosin et al., 2022; Tosin et al., 2023). Furthermore, by leveraging the high-dimensional data obtained for each tissue, the method showcased in this study has the potential to unlock a multitude of additional quality parameters during fruit maturation. This capacity for rapid and precise determination could enhance fruit production’s efficiency and effectiveness while elevating the final product’s overall quality. Finally, it is worth noting that the extensive dimensionality of the data obtained for each tissue opens possibilities for identifying and characterising other components beyond those currently presented, particularly in supporting metabolomic studies.

## 5 Conclusion

This paper proposes a tomography-like system that can predict the Vis-NIR information of the internal tissue. Applying multi-block hierarchical component analysis in conjunction with PLS enables the bi-directional reconstruction of spectral information, facilitating the prediction of internal tissue spectra (skin, pulp, and seed) and the decomposition of the overall tomato spectral information into its constituent tissues.

This novel approach allows assessing tomato maturation dynamics by analysing internal tissue characteristics, offering pertinent information for precision agricultural practices. Moreover, the method can identify physiological issues related to abiotic (e.g., water stress, high temperature) and biotic (e.g., bacterial infection). These identified stressors can be integrated into multifaceted omics techniques to understand the plant’s physiological responses.

Building on successful testing in grapes this technique, demonstrates its efficacy in the complex tissue structure of tomato. Thus, the same approach could be applied to other aqueous fruits, such as blueberries. However, further work is necessary to test the applicability of this technique in other fruits, to study the dynamic of the Vis-NIR information with the internal tissues during the maturation process, and to incorporate additional analytical data for validation.

## Data availability statement

The original contributions presented in the study are included in the article/**Supplementary Material**. Further inquiries can be directed to the corresponding author.

## Author contributions

RT: Investigation, Methodology, Writing – original draft, Writing – review & editing. MC: Investigation, Methodology, Supervision, Writing – original draft, Writing – review & editing. FM-S: Methodology, Writing – review & editing. FS: Investigation, Writing – review & editing. TB: Methodology, Writing – review & editing. RM: Investigation, Methodology, Supervision, Writing – original draft, Writing – review & editing.
