# Supplementary material for: Bi-directional hyperspectral reconstruction of cherry tomato: diagnosis of internal tissues maturation stage and composition
Source: Front Plant Sci. 2024 Feb 15;15:1351958. doi: 10.3389/fpls.2024.1351958 (PMC10905776; doi:10.3389/fpls.2024.1351958)
Supplement: Supplementary file 1 [file Image_1.pdf]

Bi-directional hyperspectral reconstruction of cherry tomato: diagnosis of internal tissues maturation stage and composition

Renan Tosin[1,2] , Mario Cunha[1,2*], Filipe Monteiro-Silva[2], Filipe Santos[2], Teresa Barroso[2], Rui Martins[2]

[1] Department of Geosciences, Environment and Spatial Planning, Faculty of Sciences of the University of Porto, Rua do Campo Alegre, Porto, Portugal.

[2] INESC TEC - Institute for Systems and Computer Engineering, Technology and Science, Campus da Faculdade de Engenharia da Universidade do Porto, Rua Dr. Roberto Frias, S/N, Porto, Portugal.

*** Correspondence:**
Corresponding Author
mccunha@fc.up.pt

Supplementary Material

1 Supplementary Figures

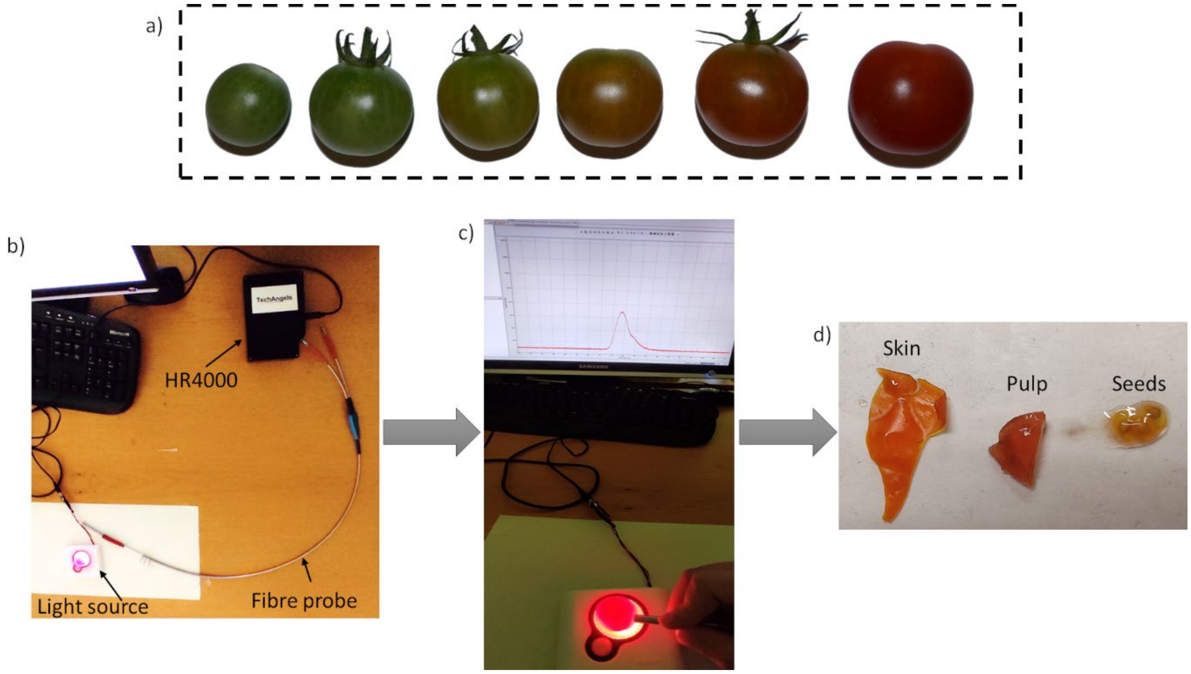

Supplementary Figure 1. Gradient of tomatoes (a) in different stages of maturation used in the experiment and the tomography-like system used to obtain the spectral information of the tomato and the internal tissues (skin, pulp and seed). In b), the spectrometer (HR4000), the fibre probe, and the

light source with the tomato are described; c) it demonstrates how the spectral information is obtained; d) shows the aliquots of the skin, pulp, and seeds considered for measurements.

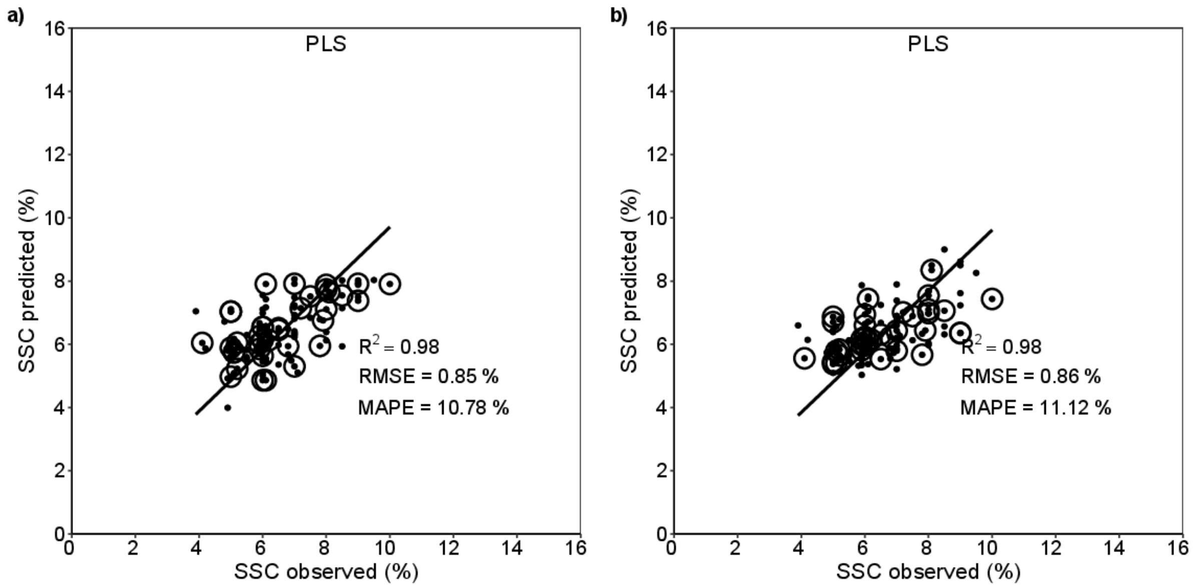

Supplementary Figure 2. Regression plots for the soluble solid content (SSC) in the original (a) and reconstructed (b) spectra considering the whole tomato. Circled observations represent the validation dataset.

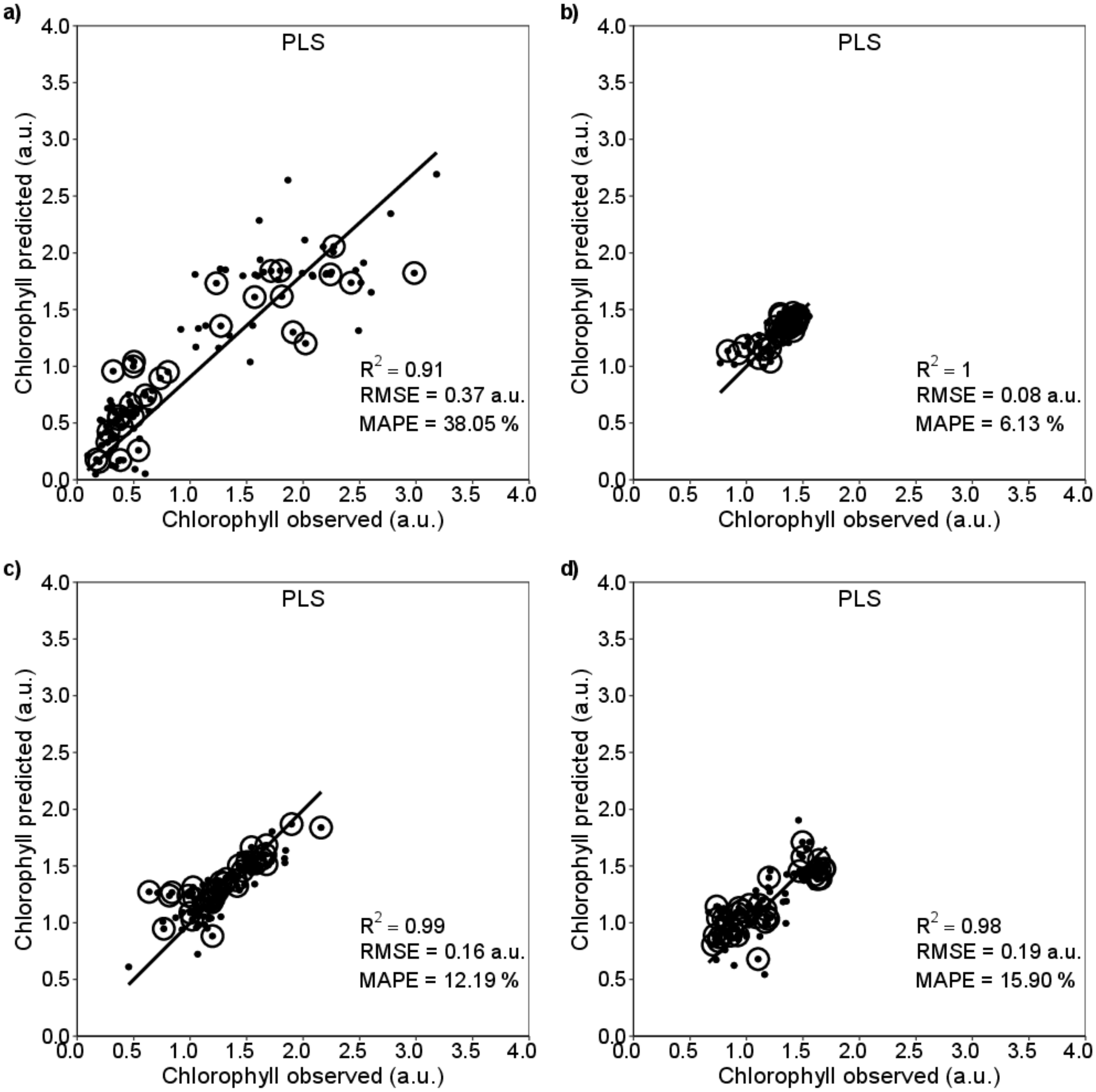

Supplementary Figure 3. Regression plots for the chlorophyll (a.u.) in the reconstructed spectra considering the whole tomato (a) and the skin (b), pulp (c) and seed (d). Circled observations represent the validation dataset.

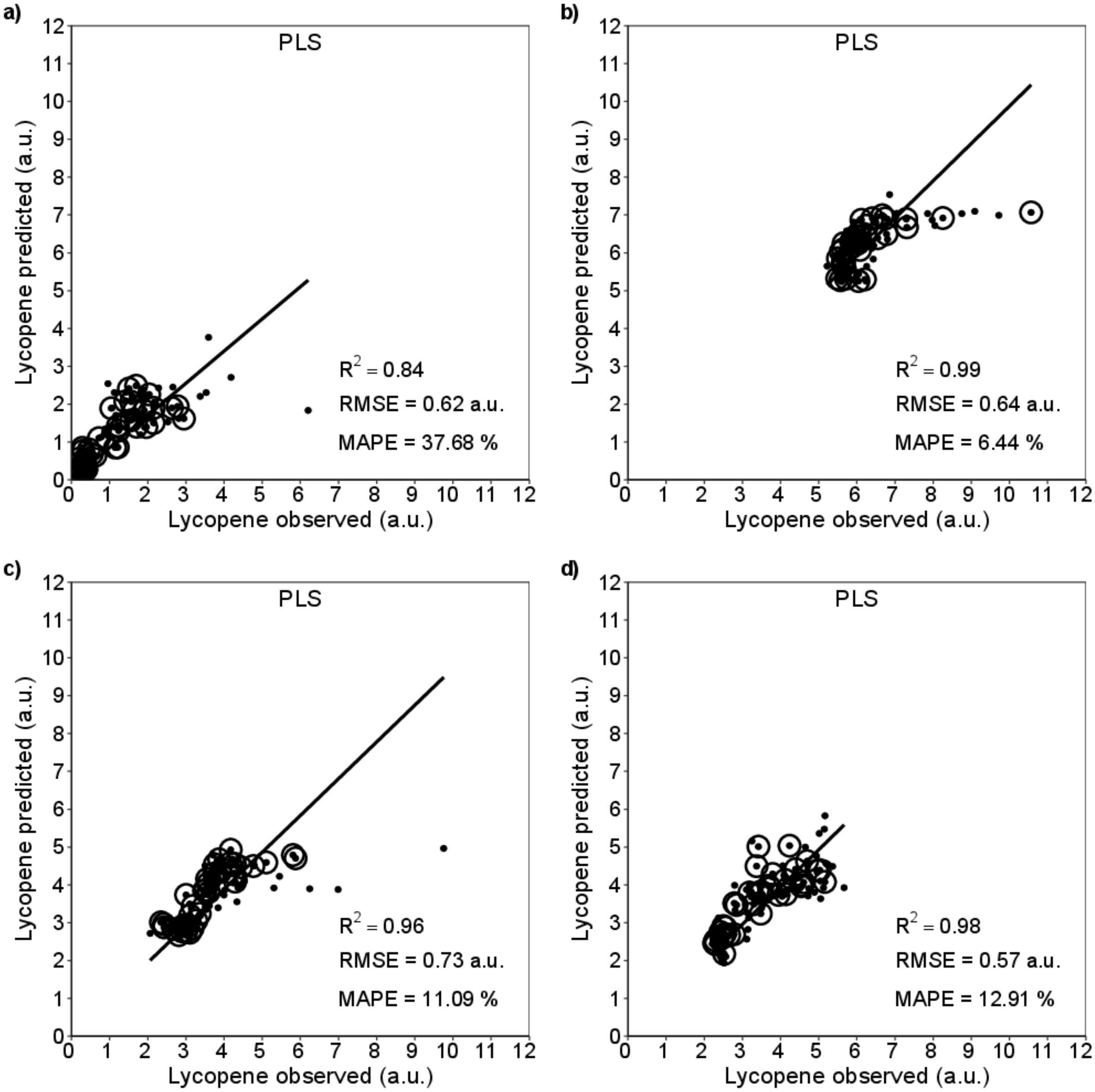

Supplementary Figure 4. Regression plots for the lycopene (a.u.) in the reconstructed spectra considering whole tomato (a) and the skin (b), pulp (c) and seed (d). Circled observations represent the validation dataset.

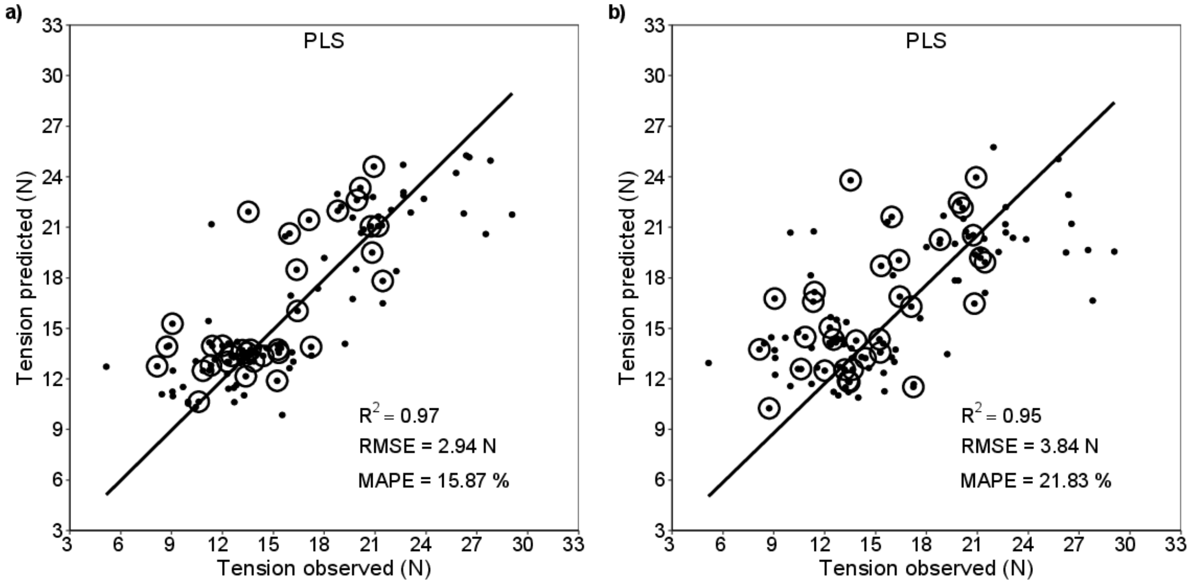

Supplementary Figure 5. Regression plots for the puncture force (N) in the original (a) and reconstructed (b) spectra considering the whole tomato. Circled observations represent the validation dataset.
